# Microstructural characteristics of fifth-century Silla gilded artifacts from Hwangnam-dong Tomb No. 120-2 in Gyeongju, Korea

**DOI:** 10.1186/s42649-026-00146-6

**Published:** 2026-10-24

**Authors:** Seonggon Kim, Euna Kim, Gyuho Kim

**Affiliations:** 1 Department of Cultural Heritage Conservation Science, Kongju National University, 56 Gongjudaehak-ro, Gongju-si, Chungcheongnam-do 32588 Republic of Korea; 2 Research Institute for Basic Science, Kongju National University, 56 Gongjudaehak-ro, Gongju- si, Chungcheongnam-do 32588 Republic of Korea

**Keywords:** Silla, Hwangnam-dong Tomb No. 120-2, Mercury-amalgam gilding, Silver-gilt, Microstructure, SEM–EDS

## Abstract

This study investigated the gilding microstructures and manufacturing techniques of a fifth-century Silla horse-harness fitting excavated from the grave-goods compartment of Hwangnam-dong Tomb No. 120-2 in Gyeongju, Korea. The specimen comprised two structural components: a gilded copper plate and a connecting iron rivet wrapped in gilded silver sheet. A single polished cross section prepared from the artifact was examined by stereomicroscopy, reflected-light metallurgical microscopy, scanning electron microscopy, and energy-dispersive X-ray spectroscopy. Residual Hg in the gilded regions of both components supports the use of mercury-amalgam gilding. Gilding-related microstructures survived on both faces and at the bent ends of the silver sheet, consistent with gilding before wrapping the rivet. EDS analysis detected Au and Hg in the amalgam regions. High-magnification imaging revealed granular remnants at both ends of the rivet that may represent Au–Hg amalgam residues associated with incomplete finishing. Local gilding thicknesses at two locations on the copper plate were 1.33 ± 0.53 μm and 1.01 ± 0.23 μm, whereas the bright gilded regions on opposite faces of the silver sheet measured 3.69 ± 1.04 μm and 3.52 ± 0.63 μm. These measurements document local thickness differences between gilded regions on copper and silver substrates within one cross section. Together, the terminal granular remnants and substrate-associated differences in local gilding thickness provide evidence for reconstructing the gilding sequence and finishing practices employed in fifth-century Silla metalworking.

## Introduction

Metal heritage objects retain superimposed traces of manufacture, use, and corrosion during burial. In gilded objects, damage to the surface layer and the accumulation of corrosion products can obscure the original materials and assembly, making manufacturing techniques difficult to identify from external appearance alone. Microscopy reveals the spatial relationships among surviving metal, gilding layers, cracks, and pores. Correlating cross-sectional microstructures with chemical compositions allows surface treatments and constituent components to be distinguished.

Korean studies have combined microscopic observations with compositional analysis to investigate goldworking and surface decoration. In a study of Baekje gold beads, stereomicroscopy and nondestructive compositional analysis distinguished fabrication features and allowed gold purity to be compared among morphological types (Kim et al. 2022). Optical microscopy and SEM–EDS of the wooden seated Shakyamuni at Bulamsa Temple in Namyangju distinguished gold-leaf and particulate surface finishes and resolved successive gold-leaf and lacquer strata (Kim et al. 2024). These examples demonstrate how material characterization and structural observations provide complementary evidence for interpreting manufacture and later surface treatments.

Mercury-amalgam gilding uses gold and mercury to form a gold coating on a metal surface. Residual Hg and the microstructure of the coating are key indicators of this technique. Layered, granular, and porous gilding microstructures have been reported in copper-based artifacts (Ma et al. 2025). Experimental comparisons have also shown that the distribution of residual Hg differs between silver and copper substrates, demonstrating the need to examine the coating and its substrate together (Margreiter et al. 2022).

**Fig. 1:**
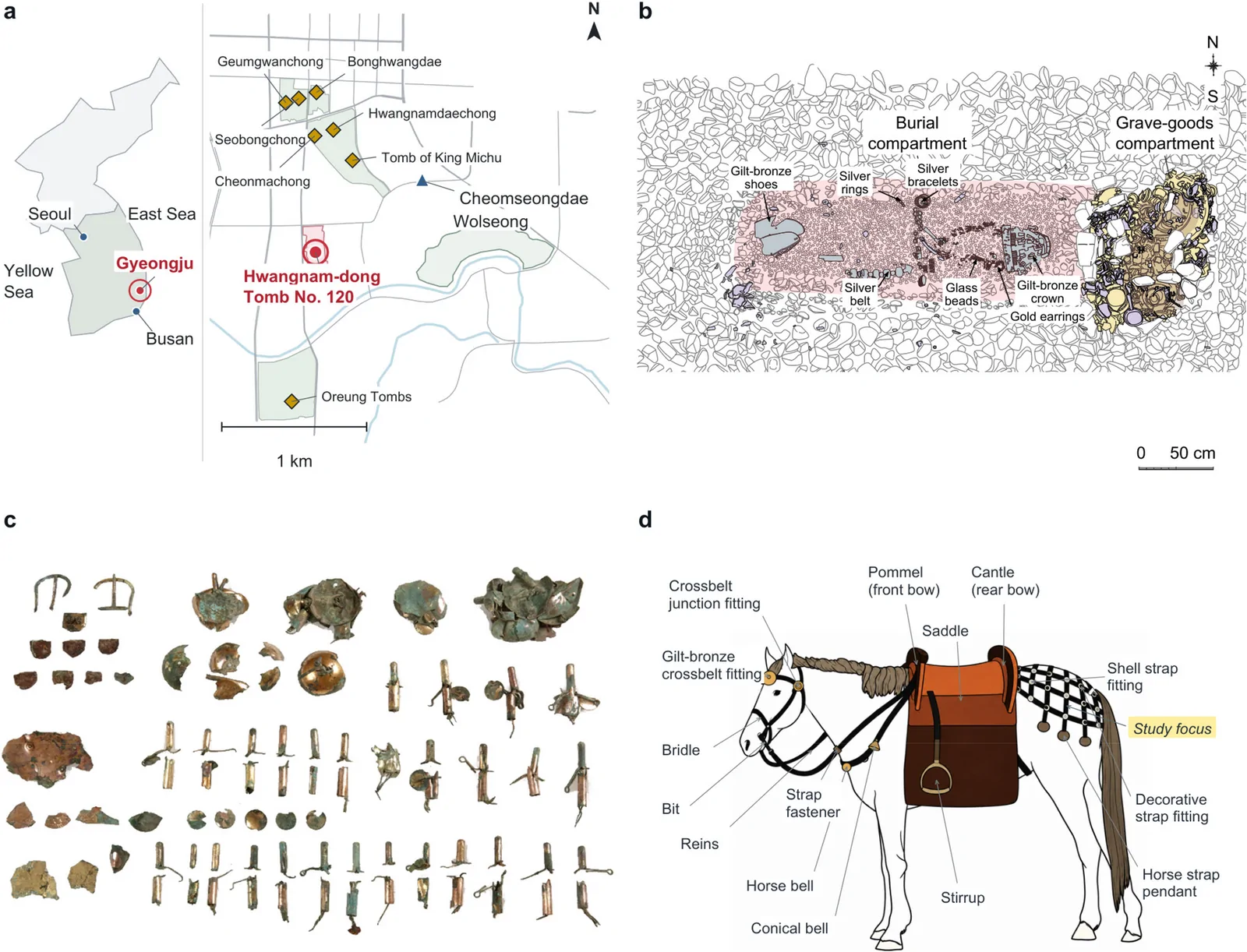
Site location and horse-harness fittings. **a** Location of the Hwangnam-dong Tomb No. 120 area in Gyeongju. **b** Plan of the burial chamber and grave-goods compartment of Tomb No. 120-2, showing the arrangement of major grave goods. **c** Assemblage of gilt-bronze horse-harness fittings from the grave-goods compartment. **d** General arrangement of horse-harness components and the functional position of the rivet examined in this study. The rivets and decorative plates were recovered from the grave-goods compartment

The Hwangnam-dong tomb complex lies in the center of the former Silla royal capital, Gyeongju. The study site is located around Hwangnam-dong Tomb No. 120, south of Daereungwon (Fig. 1a). Tomb No. 120 was known by a number assigned during the Japanese colonial period. Excavations began in May 2018, and Tombs Nos. 120-1 and 120-2, constructed by cutting into part of the mound of Tomb No. 120, were identified in 2019 (Cultural Heritage Administration 2020; Kim 2021). In Tomb No. 120-2, ornaments including a crown, earrings, glass beads, and a silver belt were arranged in the burial chamber, while horse-harness fittings, including gilt-bronze saddle bows and strap-junction fittings (unju), were recovered from a separate grave-goods compartment (Fig. 1b, c; Kim 2021). This study examines one composite fitting, consisting of a gilded copper plate and its attached rivet, from that compartment. These components decorated and secured harness-strap junctions (Fig. 1d). Regarding the chronology of the site, the personal ornaments worn by the deceased were considered to have been produced in the fifth century (Cultural Heritage Administration 2020; Kim 2021).

This study aims to characterize the gilding microstructures and manufacturing techniques of a composite Silla horse-harness fitting comprising a gilded copper plate and a connecting iron rivet wrapped in gilded silver sheet. Microscopic observations and chemical analyses of cross section are used to examine the gilding sequence, the possible relationship between terminal granular remnants and finishing, and local differences in gilding thickness between copper and silver substrates.

## Methods

**Fig. 2:**
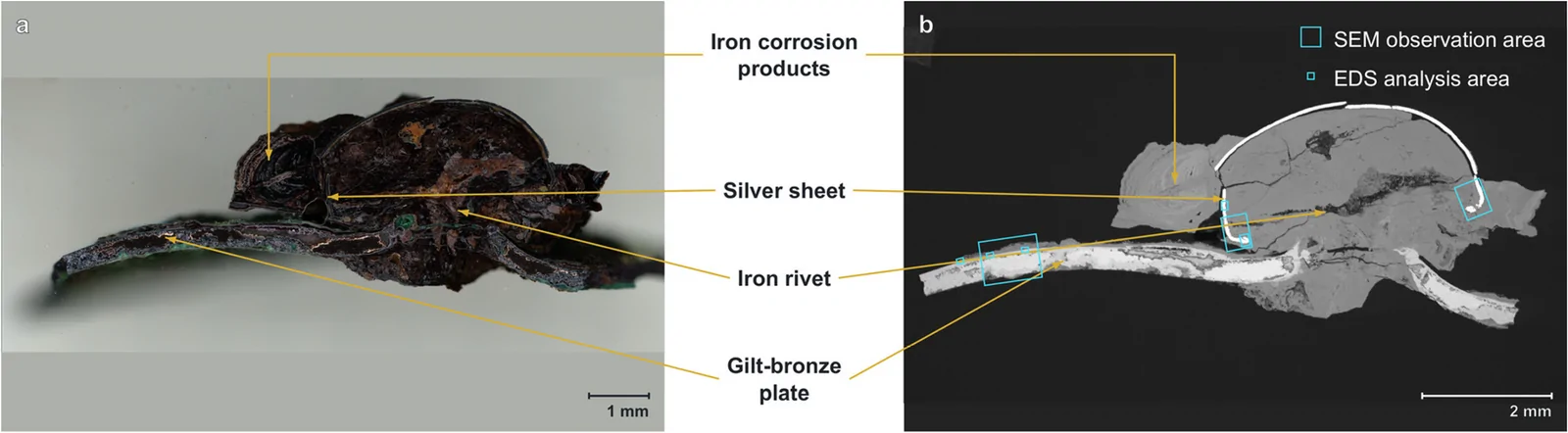
Overall structure of the single polished cross section of the horse-harness fitting. **a** Stereomicroscope image. **b** Backscattered-electron SEM image. Common labels and paired arrows identify corresponding iron corrosion products, silver sheet, iron rivet and gilt-bronze plate. Large boxes in (**b**) indicate SEM observation areas; small boxes indicate EDS analysis locations, including the terminal granular region. The small boxes are schematic location markers, enlarged where necessary for visibility, and do not represent exact EDS acquisition footprints. Scale bars: (**a**) 1 mm; (**b**) 2 mm

The study examined one composite horse-harness strap-junction fitting (unju) excavated from the grave-goods compartment of Hwangnam-dong Tomb No. 120-2, Gyeongju. One analytical specimen containing the joined decorative copper plate and iron rivet with its silver covering was prepared as cross section (Fig. 2).

### Microstructural observations

The specimen was mounted in epoxy resin and ground sequentially using P200, P400, P600, P1200, P2200, and P4000 abrasives, followed by final polishing with 6 μm and 1 μm diamond suspensions, which served as the polishing medium. At each stage, three ultrasonic cleaning cycles of 5 min each in distilled water were used to remove powdered contamination and polishing residues.

The overall cross-sectional morphology was examined by stereomicroscopy, and detailed microstructures were investigated using a metallurgical microscope (NM910, Nexcope). After optical examination, the polished section was carbon-coated for SEM observation. Backscattered electron (BSE) images were subsequently acquired using a scanning electron microscope (SEM; MIRA3-LMH, TESCAN) at an accelerating voltage of 20 kV. Reflected-light metallurgical micrographs and BSE images were compared to identify component arrangements, bending of the silver sheet, coating continuity and internal boundaries, granular microstructures, cracks, and pores.

Gilding thickness was measured in ImageJ using BSE images calibrated against their embedded scale bars. The boundaries of the bright gilded region were defined by clear BSE contrast with the substrate. Measurements were made perpendicular to the local interface at intact, clearly identifiable positions, excluding pores, open cracks, detached particles, and ambiguous interfaces. Ten local measurements in each image were summarized as mean ± standard deviation (SD).

### Chemical composition

Chemical compositions were determined by EDS (XFlash 4010, Bruker) using a detector attached to the SEM. Point analyses were acquired at 20 kV with a working distance of 10 mm and an acquisition time of 120 s. Point analyses of selected gilded regions were performed at 8000× magnification to facilitate precise beam placement within the gilding and distinguish the selected sites from the adjacent substrate. A nominally pure Cu reference was used to set the beam conditions, with the instrument beam-current setting at level 12. Quantitative spectra were acquired with count rates above 3000 counts s − 1. Quantification was performed in FP mode and checked by measuring reference alloys with nominal compositions of 80 wt% Au–20 wt% Ag and 70 wt% Au–30 wt% Ag. Elemental maps were acquired at 20 kV for 600 s using the Ag Lα, Au Lα, Hg Lα, and Fe Kα characteristic X-ray lines.

Magnification aided the selection of analysis sites but did not exclude X-ray contributions from adjacent materials at 20 kV. Tables 1 and 2 report the measured EDS compositions without post hoc subtraction of Cu or Ag or renormalization. The notes specify the possible contributions from adjacent substrates and the limitations on interpreting these values as isolated-layer compositions.

**Table 1:** EDS compositions of the gilded copper plate

Analyzed region	*n*	Composition (wt%)				
		Cu	Ag	Au	Hg	Pb
Copper substrate	3	99.84 ± 0.24	0.01 ± 0.01	0.01 ± 0.01	ND	0.14 ± 0.25
Locally uniform region	3	0.39 ± 0.07	1.88 ± 0.56	80.01 ± 1.09	17.71 ± 1.01	ND
Inner layer (thick gilding)	3	22.85 ± 6.61	1.48 ± 1.40	65.38 ± 6.98	10.30 ± 1.45	ND
Outer layer (thick gilding)	3	10.62 ± 0.79	0.15 ± 0.10	75.58 ± 1.04	13.65 ± 0.72	ND
Au-Hg amalgam	3	13.64 ± 4.15	0.36 ± 0.26	73.49 ± 1.92	12.51 ± 2.55	ND

Values are presented as mean ± standard deviation (SD) in wt%, calculated from unrounded source values and rounded to two decimal places. ND, not determined: no quantitative value was reported in the original EDS output. n is the number of EDS point analyses included in each regional mean and SD, not the number of independent artifacts or specimens. The values are measured EDS compositions acquired at 20 kV; contributions from the adjacent Cu substrate to the gilded and granular regions have not been independently separated. No post hoc subtraction of Cu or renormalization was applied, and the values should not be interpreted as isolated-layer compositions. Inner and outer layers denote spatial portions of a locally thick gilding layer, not separate gilding applications

**Table 2:** EDS compositions of the silver sheet and gilding on the iron rivet

Analyzed region	*n*	Composition (wt%)				
		Cu	Ag	Au	Hg	Pb
Silver sheet	3	0.25 ± 0.09	96.93 ± 0.38	2.72 ± 0.41	0.10 ± 0.18	ND
Gilding	3	0.29 ± 0.17	26.07 ± 22.36	47.94 ± 29.58	25.70 ± 7.05	ND
Au–Hg amalgam	3	0.67 ± 0.53	14.35 ± 16.24	61.86 ± 21.87	23.12 ± 5.10	ND

Values are presented as mean ± standard deviation (SD) in wt%, calculated from unrounded source values and rounded to two decimal places. ND, not determined: no quantitative value was reported in the original EDS output. n is the number of EDS point analyses included in each regional mean and SD, not the number of independent artifacts or specimens. The values are measured EDS compositions acquired at 20 kV; contributions from the adjacent Ag substrate to the gilded and granular regions have not been independently separated. No post hoc subtraction of Ag or renormalization was applied, and the values should not be interpreted as isolated-layer compositions

## Results

### Overall structure

The overall cross-section showed a gilded copper decorative plate attached to an iron rivet (Fig. 2). The rivet head was curved and covered with a silver sheet along its outer contour. Both ends of the sheet bent inward at the edges of the rivet. Although locally discontinuous, the sheet retained an outline that largely enclosed the rivet head. Corrosion products, cracks, and voids were observed within and around the iron region. Their association with the corroded iron is interpreted as evidence of deterioration during burial (Cho 2021).

A gold-plating layer distinct from the copper substrate was observed on the gilded copper plate. Gilding was also present on the surface of the silver sheet covering the rivet. The cross-section therefore revealed two joined structural components: a gilded copper plate and a connecting iron rivet wrapped in gilded silver sheet. These components were examined separately to characterize their microstructure and chemical composition.

### Gilded copper plate

**Fig. 3:**
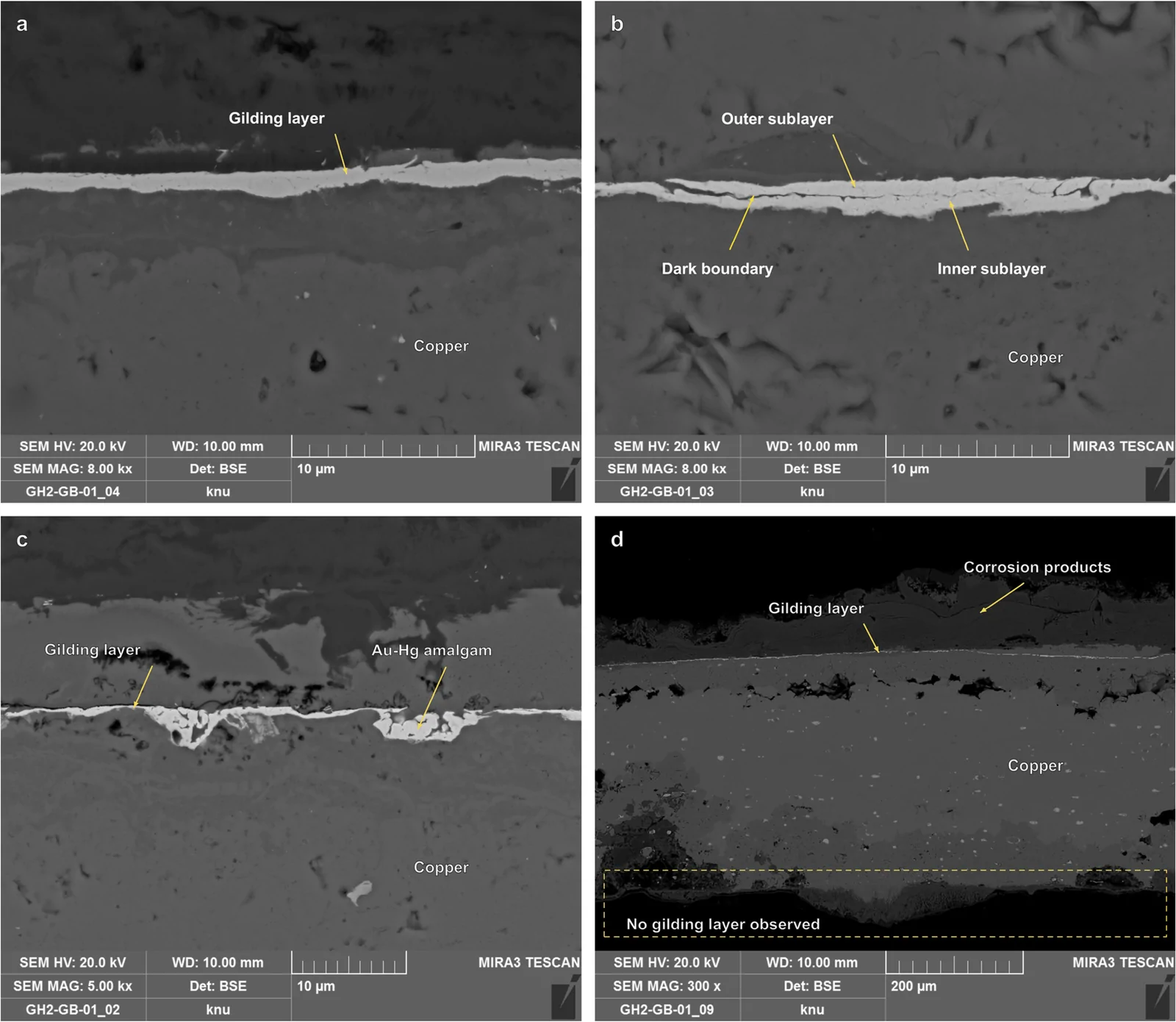
BSE images of gilding on the copper plate. **a** A locally uniform, continuous gilding band. The copper substrate contains relatively bright, compact areas and darker porous areas; this contrast may reflect local compositional differences, porosity, and surface condition. **b** A locally thick gilding layer with a dark internal boundary between the inner layer adjoining the copper substrate and the outer layer toward the surface. **c** Non-uniform gilding with irregular Au–Hg-containing microstructures. **d** The copper substrate, gilding on the upper surface, and a corroded region. No gilding layer was observed on the lower surface within the dashed box. Scale bars: (**a**–**c**) 10 μm; (**d**) 200 μm

The gilded copper plate consisted of a predominantly copper substrate and a surface gilding layer. The substrate contained a mean Cu concentration of 99.84 ± 0.24 wt% and a mean Pb concentration of 0.14 ± 0.25 wt%. Gilding extended along the upper surface of the observed cross-section, with a corroded region above it. No gilding layer was observed on the opposite surface (Fig. 3d; Table 1). Within the copper substrate beneath the bright gilding band in Fig. 3a, relatively bright, compact areas and darker, locally porous areas were observed. The BSE contrast may reflect local compositional differences as well as porosity and surface condition. Corrosion-related alteration is a possible explanation, but the contrast alone does not identify corrosion products or establish the extent of copper dissolution.

The gilding morphology varied along the same plate. Here, uniform gilding refers to a locally continuous band of relatively even thickness, whereas non-uniform gilding refers to regions with a dark internal boundary or irregular granular microstructures. These terms describe the preserved morphology. The region in Fig. 3a exhibited a relatively uniform bright band along the substrate surface. Point analyses detected Au and Hg in this region, together with Cu whose contribution from the adjacent substrate could not be separated.

In a locally thick gilding layer, a dark internal boundary occurred between the inner layer adjoining the copper substrate and the outer layer toward the surface (Fig. 3b). Both regions contained Au and Hg. The terms inner and outer identify spatial portions of this thick gilding layer and do not indicate separately applied coatings.

Parts of the gilding protruded or curved toward the substrate from the band-shaped layer (Fig. 3c). Granular microstructures that did not form a thin continuous band were observed in these curved regions. Au, Hg, and Cu were detected in these irregular microstructures; contributions from adjacent materials could not be independently resolved.

The detection of Hg together with Au in both the locally uniform and non-uniform gilded regions supports the use of mercury-amalgam gilding. The dark boundary and granular microstructures document local variations in the preserved coating; they do not establish two successive gilding applications.

### Iron rivet with silver sheet and gold plating

**Fig. 4:**
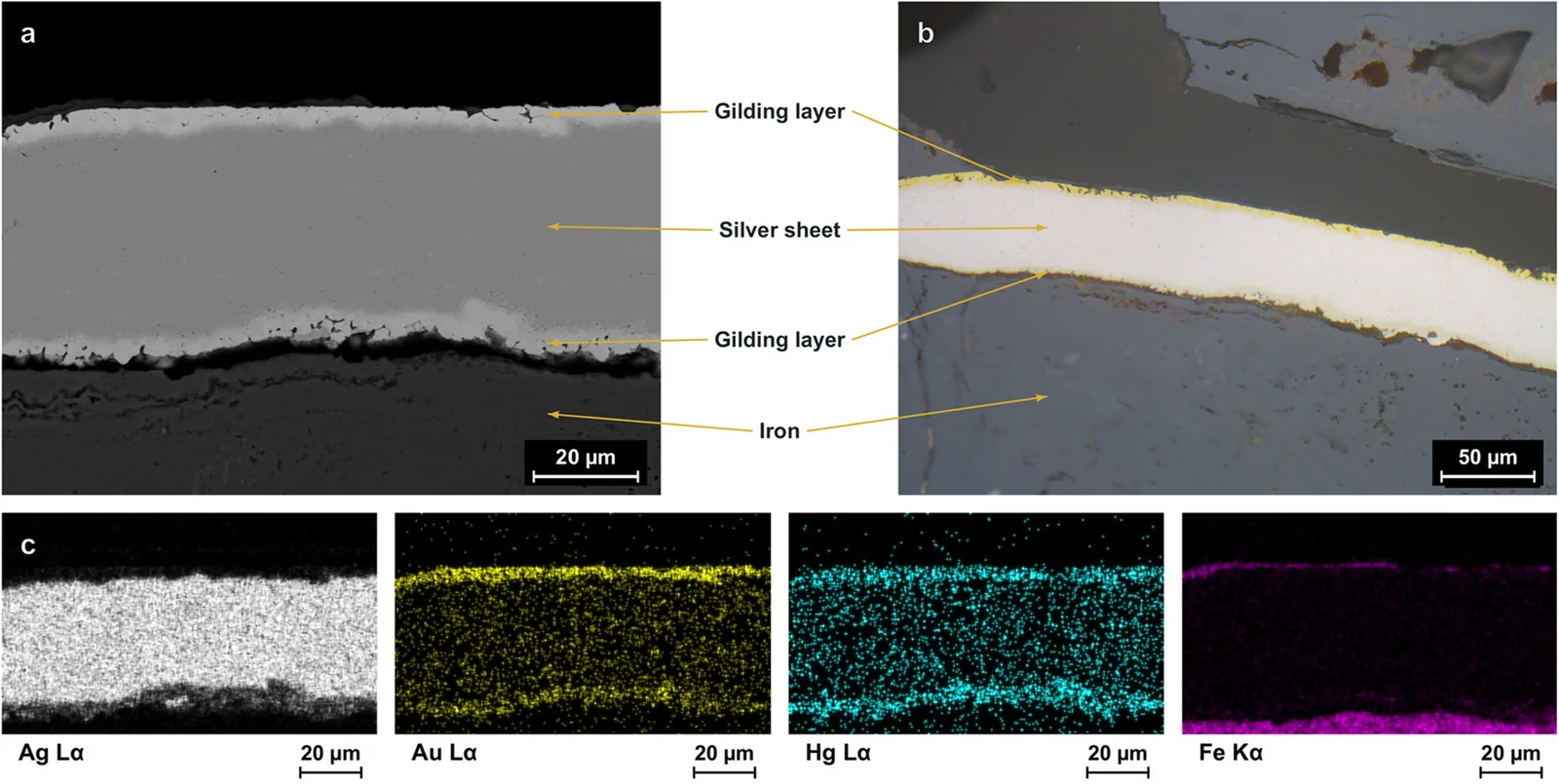
Microstructure and elemental distributions of the iron rivet with silver sheet and gold plating. **a** BSE image and (**b**) reflected-light metallurgical micrograph. Shared arrows identify the silver sheet, the gilding on both faces, and the underlying iron region. **c** EDS maps acquired using Ag Lα, Au Lα, Hg Lα, and Fe Kα, from left to right. Scale bars: (**a**, **c**) 20 μm; (**b**) 50 μm

The silver sheet covering the rivet appeared as a bright, silvery-gray plate in reflected-light metallurgical micrographs and as a continuous band distinct from the underlying iron region in BSE images (Fig. 4a, b). The interior of the sheet contained a mean Ag concentration of 96.93 ± 0.38 wt%, together with Au 2.72 ± 0.41 wt%, Cu 0.25 ± 0.09 wt%, and Hg 0.10 ± 0.18 wt%. These results confirmed that the sheet surrounding the rivet consisted predominantly of Ag (Table 2).

Gold-colored microstructures were distributed along the upper and lower boundaries of the silver sheet in reflected-light metallurgical micrographs. In BSE images, these regions appeared brighter than the sheet interior and included both continuous segments along the boundaries and discrete granules. Elemental maps showed Ag distributed throughout the sheet, whereas Au and Hg were detected in microstructures along its boundaries. The Fe signal occurred mainly below the sheet, corresponding to the arrangement of the silver covering and the underlying iron region (Fig. 4c).

Point analyses detected Au and Hg in both the gilded regions on the silver sheet and the adjacent granular microstructures.

**Fig. 5:**
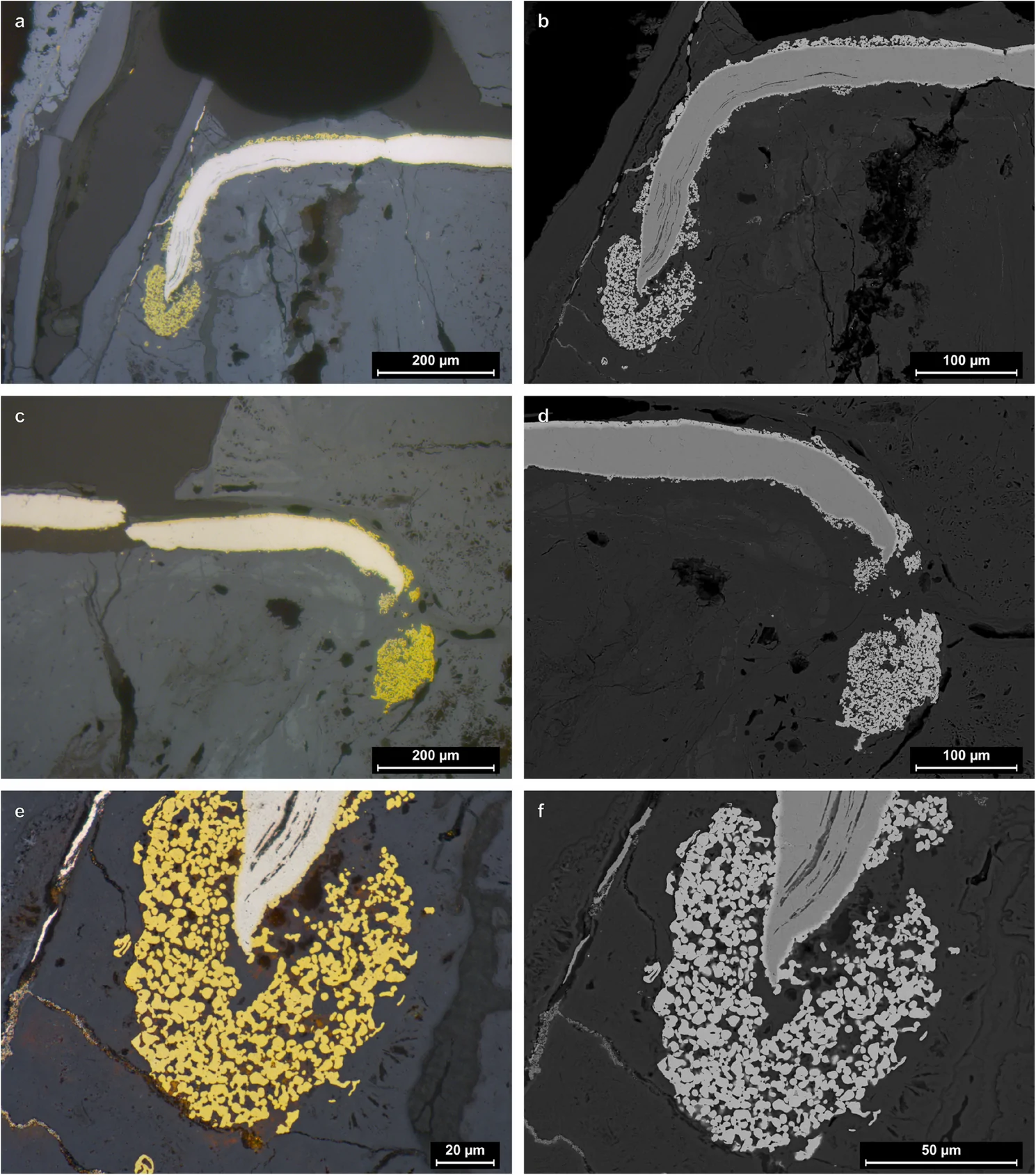
Bent silver-sheet ends and granular gilding remnants. Reflected-light metallurgical micrographs (**a**, **c**, **e**) are paired with BSE images (**b**, **d**, **f**). **a**, **b** One end of the sheet. **c**, **d** The opposite end. **e**, **f** Higher-magnification views of granular remnants around the end shown in (**a**, **b**). Scale bars: (**a**, **c**) 200 μm; (**b**, **d**) 100 μm; (**e**) 20 μm; (**f**) 50 μm

Reflected-light metallurgical micrographs showed that both ends of the silver sheet bent inward around the rivet head, with gold-colored remnants on the surfaces of the bends and around the ends (Fig. 5a, c). The corresponding BSE images showed the bent sheet and adjacent remnants (Fig. 5b, d). These remnants sometimes followed the sheet contour, whereas aggregates of granules were present beyond the ends. At higher magnification, dark voids were visible between rounded particles and irregularly interconnected particles (Fig. 5e, f). These observations revealed the shape of the silver sheet surrounding the iron region and the distribution of gilding-related remnants along its boundaries and ends. EDS point analysis was also performed on the terminal granules shown in Fig. 5e, f.

## Discussion

The repeated detection of Hg in the gilded regions of both the gilded copper plate and the silver sheet, together with the observation of granular microstructures containing Au and Hg, supports the use of mercury-amalgam gilding. Heating after amalgam application removes some of the Hg and leaves a gold-containing gilded microstructure.

Previous studies have discussed elemental migration between gilding and its substrate during heating (Jeon et al. 2010; Shao et al. 2023). In early medieval copper-based jewellery, an inner Au–Cu sublayer associated with copper interdiffusion was identified beneath the outer gilding alloy (Ottenwelter et al. 2022), while sublayers with different compositions and microstructures were reported in gilded artifacts from a Han tomb in Qufu (Ma et al. 2025). Such processes may have contributed to Cu detected in the gilded regions of the present specimen. However, the 20 kV point analyses may also include X-rays generated in the adjacent copper substrate. The present data cannot independently distinguish actual Cu migration from substrate contributions within the X-ray generation volume; Cu detection is therefore not interpreted as conclusive evidence of diffusion.

The silver sheet retained gilding on both faces, including the face directed toward the iron rivet, and gilding-related remnants extended around the inward-bent ends. This distribution supports a manufacturing sequence in which the sheet was gilded before being wrapped around the rivet. In this interpretation, a previously gilded sheet provided the decorative covering of an iron structural component that joined the copper plate. The observed relationships thus link surface treatment with assembly in a single composite Silla fitting.

The porous granular remnants at the bent ends of the silver sheet around the rivet head may represent Au–Hg amalgam residues associated with incomplete burnishing or finishing. Recessed regions of the rounded rivet head would have been less accessible to finishing tools, and the small residual particles resolved by high-magnification microscopy may have been difficult to recognize by unaided inspection. This is a possible explanation for their location and morphology, rather than proof of an omission by the maker.

**Fig. 6:**
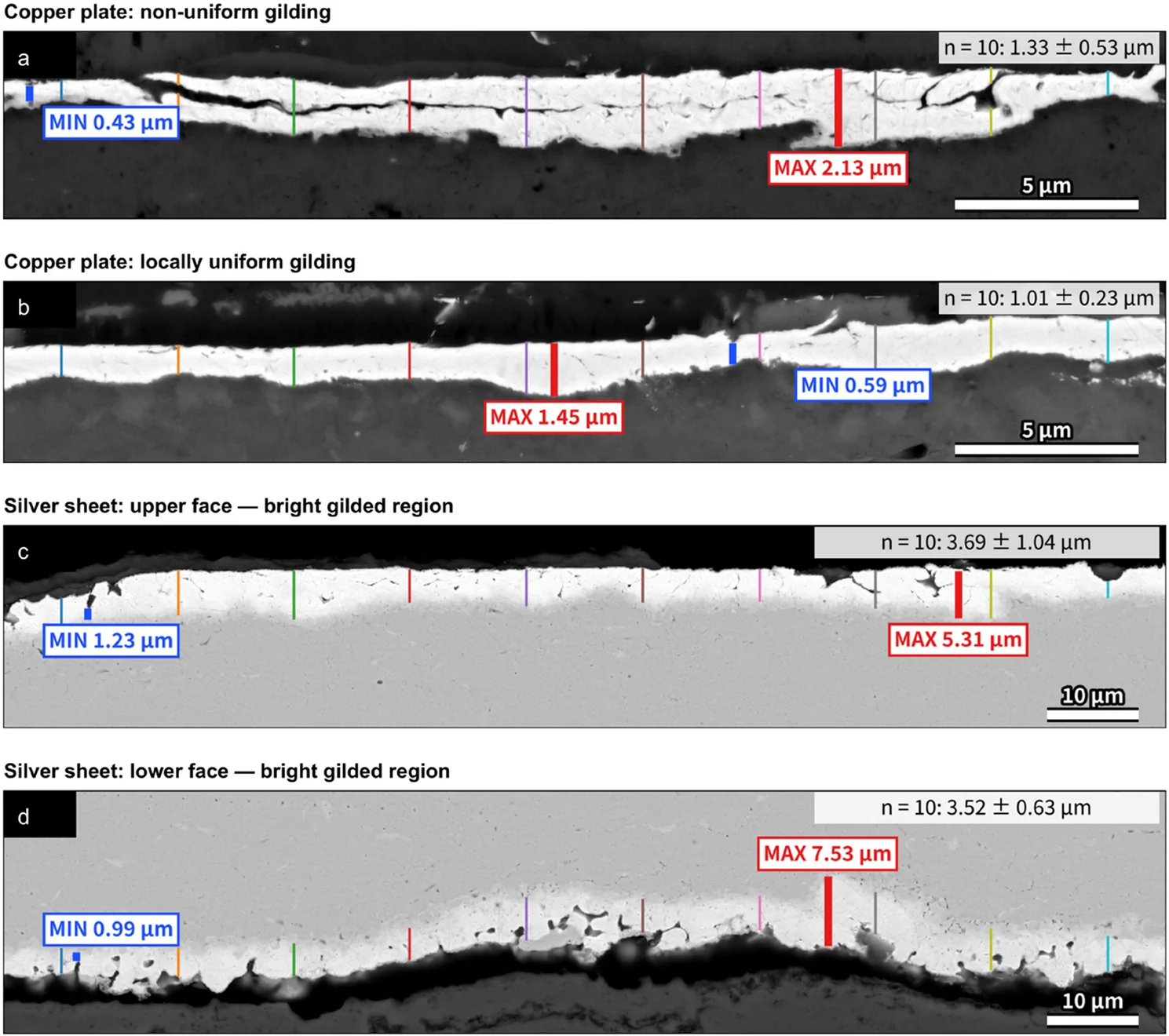
Gilding thickness in selected regions on different substrates. **a**, **b** Non-uniform and locally uniform gilded regions on the copper plate, respectively. **c**, **d** Bright gilded regions on the upper and lower faces of the silver sheet. Thin colored lines indicate the 10 local positions used to calculate each mean ± SD; they represent variation within one image, not independent specimens. Red and blue markers indicate maxima and minima from a separate search across the full image width using a five-column median. Scale bars: (**a**, **b**) 5 μm; (**c**, **d**) 10 μm

BSE images were used to compare gilding thicknesses on the two substrate metals. The gilding thicknesses measured in two regions of the gilded copper plate were 1.33 ± 0.53 μm and 1.01 ± 0.23 μm, respectively, whereas the bright gilded regions on the upper and lower faces of the silver sheet measured 3.69 ± 1.04 μm and 3.52 ± 0.63 μm, respectively (Fig. 6). The measured regions on the silver sheet were approximately 2.65–3.65 times as thick as those on the gilded copper plate; however, this ratio represents differences between the selected observation sites. Previous studies reported mean gilding thicknesses of 2.2–25.2 μm for copper-based artifacts from a Han tomb in Qufu (Ma et al. 2025) and approximately 4–6 μm for coronet ornaments with a pure copper substrate excavated in Xi’an (Shao et al. 2023). The regional mean thicknesses of the gilding on the gilded copper plate were below both reported ranges. The regional means for the silver-sheet gilding were below the range reported for the Xi’an ornaments but within the wider range reported for the Qufu artifacts.

## Conclusions

The Silla horse-harness fitting excavated from the grave-goods compartment comprised a gilded copper plate and a connecting iron rivet wrapped in gilded silver sheet. Microscopy resolved the silver covering, its inward-bent ends, gilding on both faces, and terminal granular remnants. EDS analyses examined the sheet and gilding regions as well as the terminal granules.

The detection of Hg together with Au in the gilding on the gilded copper plate and the silver sheet supports the use of mercury-amalgam gilding. The gilded copper plate exhibited locally uniform bands and non-uniform regions characterized by a dark internal boundary or granular microstructures. These morphological variations do not establish the number of gilding applications.

The bright gilded regions on the silver sheet were thicker than the measured gilding on the copper plate, with ratios between regional means of approximately 2.65–3.65. Gilding on both faces and at the bent ends of the silver sheet is consistent with gilding before wrapping the rivet. High-magnification imaging revealed terminal granular remnants that may represent Au–Hg amalgam residues associated with incomplete finishing, while later alteration and preparation effects remain possible explanations. Together, the terminal remnants and substrate-associated differences in local gilding thickness provide microstructural evidence for reconstructing the gilding sequence and assessing finishing practices in this Silla fitting.

## Data Availability

The data and materials used and analyzed in this study are available from the corresponding authors upon reasonable request.

## Abbreviations

BSE: Backscattered electron
EDS: Energy-dispersive X-ray spectroscopy
SEM: Scanning electron microscopy
SD: Standard deviation
ND: Not determined (no quantitative value reported in the source data)
